# Young's Dietary in Typhoid Fever Cases

**Published:** 1909-12-11

**Authors:** 


### Dietetics.

### YOUNG'S DIETARY IN TYPHOID FEVER CASES.

There are observers who hold that medical treatment in typhoid fever may in many cases be entirely omitted. Few believe that the dietary is unimportant. According to Young, troublesome symptoms during typhoid fever can be reduced to a minimum if the diet which is allowed complies with the following requirements:

1. It must be such that no solid residue, and certainly none of any irritating character, enters that part of the intestinal tract where the local lesions are situated.

2. It must be such that fermentation of a kind to generate flatus does not take place.

3. Inasmuch as the whole of the digestive functions are below par it must be one which is readily digested and assimilated.

4. It must be such that the various tissues are provided with proper material for the renewal of that waste of substance and vitality common to all prolonged fevers, and especially such pyrexial conditions as are accompanied by the circulation of toxins.

In a properly dieted case constipation takes the place of diarrhœa, but this tendency can be corrected by the use of an enema every other morning. Young mentions a list of foods devised to satisfy the craving of the enteric patients for an ampler dietary:

1. Benger's and Mellin's foods, made with or without milk and fortified with cream, are of temporary value. So are jellies, bread crumbs, isinglass or rusks in beef-tea, and light puddings.

2. Bread jelly made by thoroughly soaking stale bread, pressing out the water, and allowing the pulp to simmer gently for one or two hours. Strain through muslin and allow the filtrate to set. Two tablespoonfuls of the jelly suffice for one feed.

3. Raw meat pulp, carefully prepared and given in the form of little balls, to be eaten with a rusk.

4. Junket made in the usual way and given, if desired, with cream or brandy.

5. Suet puddings, given after the patient's temperature has been normal for a few days. The suet must be shredded in thin slices and all the fibre removed. Wheat flour, with an equal quantity of maize, should be used. The latter contains little gluten. Cook well and serve with sweet sauce or gravy.

6. Fish, best in the form of whiting.

7. Modified milk diets: To prevent alteration in flavour and formation of scum the following directions for boiling the milk should be followed:

(i.) Use an ordinary double milk pan, or a smaller covered saucepan containing the milk placed inside a larger one containing the water.

(ii.) Let the water in the outer pan be cold when placed on the fire.

(iii.) Bring the water up to the boiling point, and maintain it at this for three or four minutes without removing the lid of the inner milk pan.

(iv.) Cool the milk down quickly by placing the inner pan in one or two changes of cold water without removing the lid.

(v.) When cooled down, aerate the milk by stirring well with a spoon.

